# FDA-Approved Small Molecules in 2022: Clinical Uses and Their Synthesis

**DOI:** 10.3390/pharmaceutics14112538

**Published:** 2022-11-21

**Authors:** Davide Benedetto Tiz, Luana Bagnoli, Ornelio Rosati, Francesca Marini, Claudio Santi, Luca Sancineto

**Affiliations:** Group of Catalysis, Synthesis and Organic Green Chemistry, Department of Pharmaceutical Sciences, University of Perugia, Via del Liceo 1, 06123 Perugia, Italy

**Keywords:** FDA, drugs, synthesis, new therapies

## Abstract

This review describes the recently FDA-approved drugs (in the year 2022). Many of these products contain active moieties that FDA had not previously approved, either as a single ingredient or as part of a combination. These products frequently provide important new therapies for patients with multiple unmet diseases. The diverse small molecules are described according to the date of approval and their syntheses is discussed. This review comprises classical chemical scaffolds together with innovative drugs such as a deuterium-containing drug.

## 1. Introduction

The constant research for innovative therapies leads every year to molecules that are approved by the United States Food and Drug Administration (FDA), a federal agency of the U.S. Department of Health and Human Services.

Novel building blocks and their connections have been explored much in recent years allowing drug hunters to explore a much bigger chemical space [1].

Small molecule drugs are organic compounds with low molecular weight. For a long time, they have been the backbone of the pharmaceutical industry. There are several benefits that make small molecules important drugs in therapy, such as the possibility of oral administration and cell membrane permeability which allow them to reach specific tissue and targets. For this purpose, small molecule drugs can be designed in order to acquire, for example, specific affinity and selectivity (target, tissue penetration and distribution) [2].

The goal of this review is to highlight the chemical entities approved in the year 2022 for clinical use. The approved drugs (and their synthesis) are listed according to their chronological approval date [3]. This paper focuses specifically on small molecules, as high-molecular weight peptides, vaccines and other biotechnological drugs are beyond the scope of this work. Interestingly, from the perspective of the functional group characterizing the molecules herein discussed, four contain a sulfonamide functionality, one is a macrocycle and two are steroidal structures. Two radioisotope/contrast agents and one deuterium-containing drug were approved. These latter surely represent a very innovative starting point for future drug discovery efforts. Halogenated (mainly chlorinated and fluorinated) drugs still characterize a consistent percentage of the approved drugs, underlining the importance of halogens in drug discovery [4]. All the structures contain at least one aromatic ring except for two drugs which possess steroid-like structure.

## 2. FDA-Approved Drugs in 2022

### 2.1. Daridorexant

**Scheme 1:**
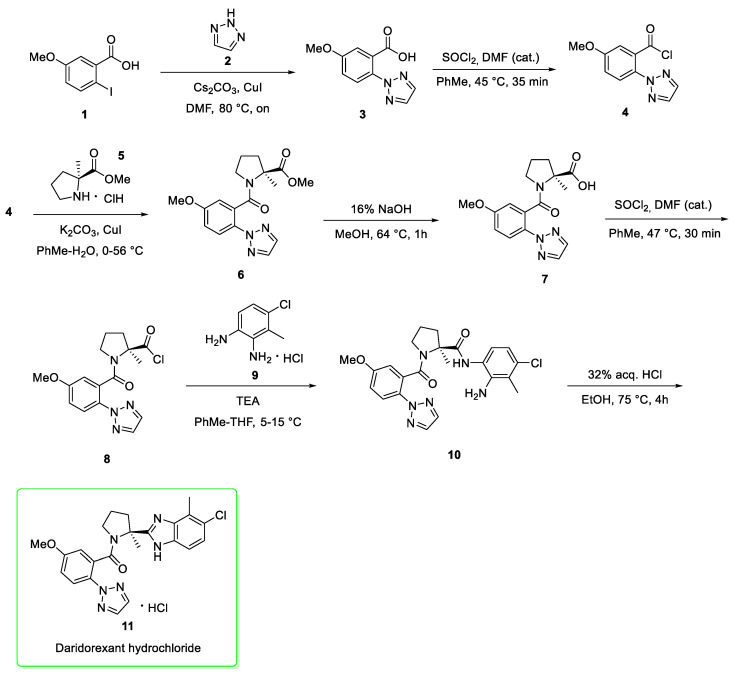
Synthesis of daridorexant hydrochloride (**11**) [8].

Approved at the beginning of 2022 (7 January 2022) and launched by Idorsia U.S. [5], daridorexant (**11**) (brand name Quviviq^®^) is a 1,2,3-triazole derivative with an expected global sales to 2026 of $1052 million [6]. The mechanism of action of daridorexant in the treatment of insomnia is presumed to be through antagonism of orexin receptors. The orexin neuropeptide signaling system plays a role in wakefulness [7]. The synthesis [8] is reported in Scheme 1, and when performed at the kilogram-scale it has moderate yield: 50% [8].

The first step involves the reaction between 2-iodo-5-methoxybenzoic acid (**1**) and 1,2,3-triazole (**2**) in a copper-mediated Ullmann-like coupling reaction that affords the triazole derivative **3**. After the activation of carboxylic acid by thionyl chloride to generate the acyl chloride **4**, the amidation with the (*S*)-proline derivative **5** afforded the amide **6.** The alkaline hydrolysis of **6** provided the carboxylic acid **7**, in turn converted into the corresponding acyl chloride **8** which upon treatment of the 4-chloro-3-methylbenzene-1,2-diamine (**9**) in presence of triethylamine gave the bis-amide **10.** The treatment of **10** with hydrochloric acid afforded Daridorexant (**11**) in the form of hydrochloric salt.

### 2.2. Abrocitinib

**Scheme 2:**
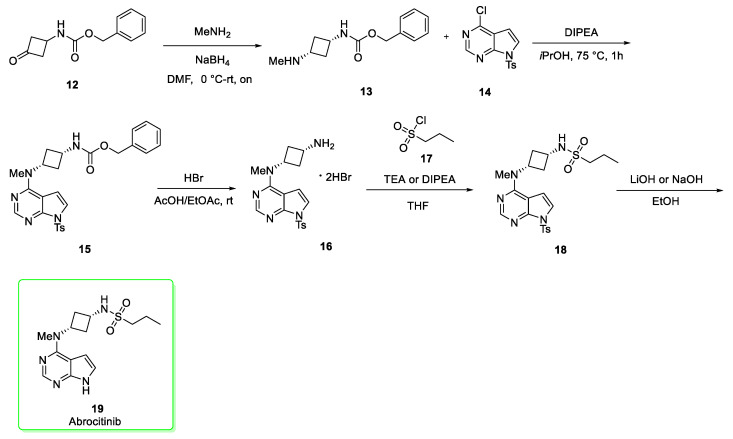
Synthesis of abrocitinib (**19**) [10].

Approved in January 2022 (14 January 2022), abrocitinib (brand name Cibinqo^®^) is a selective Janus kinase 1 (JAK1) inhibitor effective and safe for the treatment of atopic dermatitis (AD), with good oral bioavailability as well as lack of immunogenicity, addressing some of the limitations of biologic drugs currently available. It is expected that the global sales forecast in 2026 will be $760 million [6]. Abrocitinib was developed by Pfizer to treat moderate to severe AD patients [9]. Its synthesis (Scheme 2) [10] begins with the reductive amination between benzyl (3-oxocyclobutyl)carbamate and methylamine to afford diasteroselectively the desired *cis* cyclobuthyl amine derivative **13**. A high selectivity and purity biocatalityc method to obtain amine **13** was later reported in a green-fashion [11]. Derivative **13** was nucleophilically added to the tosyl-protected pyrrolo [2,3-*d*]pyrimidine **14** (the electron-withdrawing effect of the tosyl group allowed the nucleophilic aromatic substitution reaction to occur readily in high yield) in the presence of diisopropylethylamine (DIPEA) to give the intermediate **15** which underwent the deprotection of carboxylbenzyl protecting group under acidic conditions (HBr) to afford **16**. The free primary amino group of **16** reacted with the propane-1-sulfonyl chloride **17** to provide the sulfonamide **18**. The last stage (yield: 74%) involved the removal of tosyl group by using lithium hydroxide or sodium hydroxide in ethanol to generate Abrocitinib (**19**).

### 2.3. Mitapivat

**Scheme 3:**
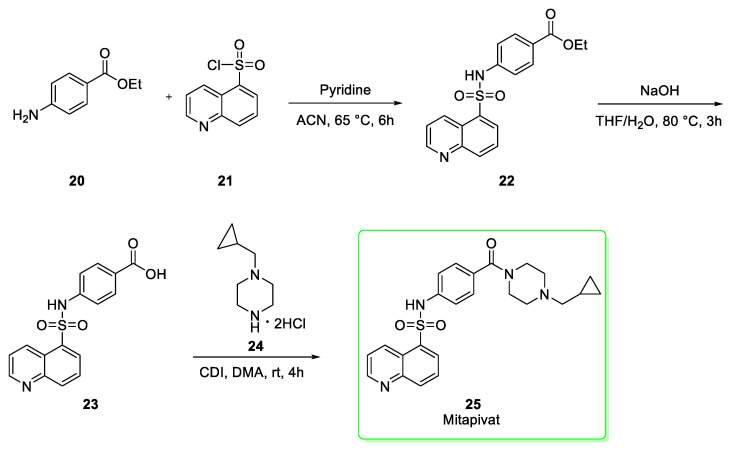
Synthesis of mitapivat (**25**) [13].

Approved in February 2022 (17 February 2022) and developed by Agios Pharmaceuticals, mitapivat (**25**, brand name Pyrukynd^®^) was approved for the treatment of hereditary hemolytic anemias [12]. It is expected that the global sales forecast in 2026 will be $511 million [6]. This molecule is an allosteric activator of the pyruvate kinase enzyme and it is chemically a sulfonamide. Its synthesis [13], reported in Scheme 3, begins with ethyl-4-aminobenzoate (**20**) that nucleophilically attacks, under alkaline conditions in pyridine, the quinoline-based sulfonyl chloride (**21**) to afford the sulfonamide intermediate **22**. After the alkaline hydrolysis the carboxylic acid **23** was obtained and then amidated using 1,1′-carbonyldiimidazole (CDI) and (cyclopropylmethyl)piperazine dihydrochloride (**24**) in dimethylacetamide (DMA) to give mitapivat (**25**); in this last step the yield reported was 90% [13].

### 2.4. Pacritinib

Pacritinib (**36**, brand name Vonjo^®^) was authorized (28 February 2022) for the treatment of high-risk primary or secondary myelofibrosis in adults with low platelets [14].

Discovered and synthesized by S*BIO Pte Ltd. (Singapore), it is a Janus kinase 2 (JAK2) inhibitor [15] with an expected sales forecast in 2026 of $496 million [6].

**Scheme 4:**
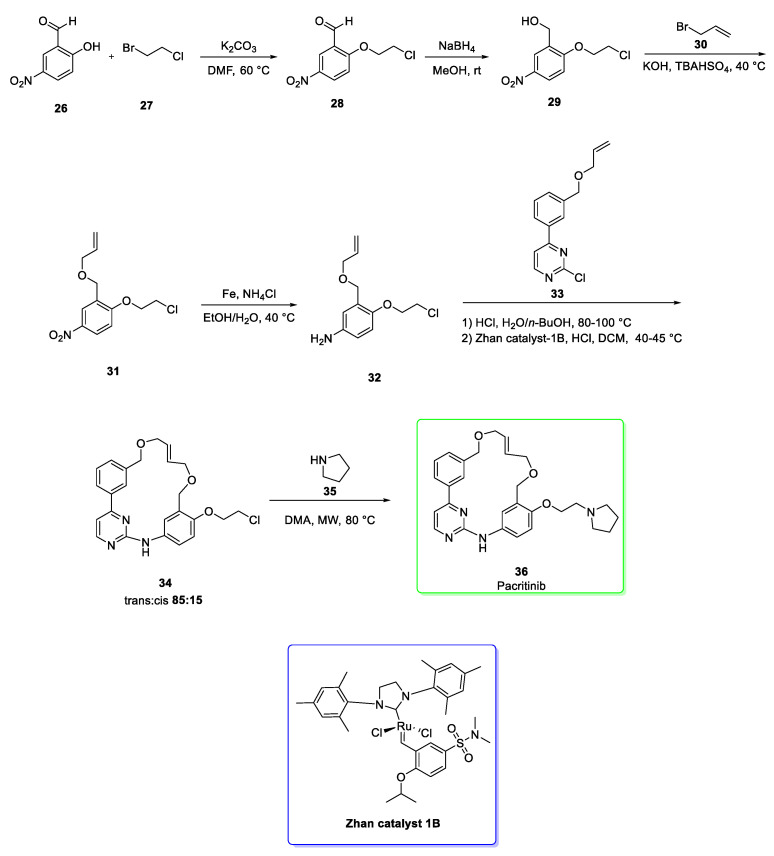
Synthesis of pacritinib (**36**) [16] and the structure of Zhan catalyst (blue box).

The synthesis of pacritinib (Scheme 4) [16] starts with the nucleophilic attack of 2-hydroxy-5-nitrobenzaldehyde (**26**) to 1-bromo-2-chloroethane (**27**) in the presence of potassium carbonate to afford the aromatic ether **28**. The reduction of the aldehydic group of **28** afforded the primary alcohol **29** which was then subjected to the reaction with allyl bromide **30** in the presence of tetrabutylammonium hydrogensulfate (TBAHSO_4_) to give the diether **31**. The nitro group of **31** was reduced to amine under iron/ammonium chloride conditions to afford the amine **32**. After the nucleophilic aromatic substitution, the ring-closing metathesis (RCM) between **32** and the chloropyrimidine **33** afforded the macrocycle **34** via ruthenium-based approach using the Zhan catalyst 1B. The macrocycle was obtained as inseparable mixtures of approximately 85:15 *trans:cis* geometry [16]. Pacritinib (**36**) was eventually converted via nucleophilically addition of pyrrolidine **35** to the chloride **34**; in this step a 83% yield was reached [16].

### 2.5. Ganaxolone

Approved in March 2022 (18 March 2022), ganaxolone (**41**) was authorized for the treatment of seizures in cyclin-dependent kinase-like 5 deficiency disorders [3]. The mechanism of action of Ganaxolone is yet unknown. Most probably it modulates (positive allosteric modulator) both synaptic and extrasynaptic GABA_A_ receptors to normalize over-excited neurons [17,18].

It is expected that the global sales forecast in 2026 will be $434 million [6].

**Scheme 5:**
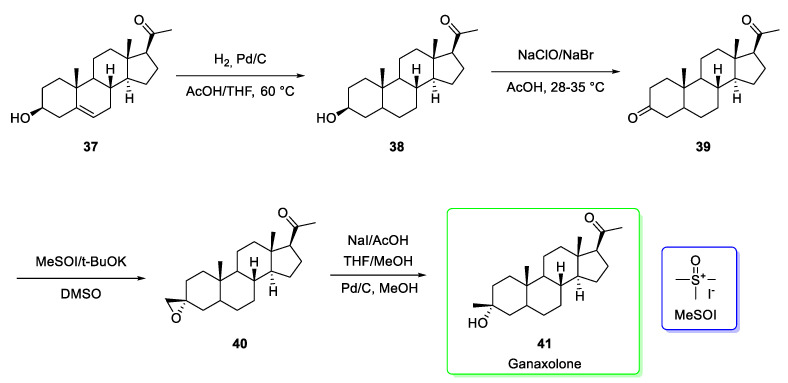
Synthesis of ganaxolone (**41**) [19] and the structure of trimethylsulfoxonium iodide (MeSOI, blue box), the agent used to introduce epoxide ring.

Developed by Marinus Pharmaceuticals, ganaxolone (brand name Ztalmy^®^) possesses a steroidal structure and its synthesis [19] (Scheme 5) starts from the precursor pregnenolone **37**. The initial chemoselective hydrogenation catalyzed by palladium on carbon afforded **38** which is oxidized under hypochlorite conditions to afford the corresponding ketone **39**.

In the following steps, the epoxide formation mediated by trimethylsulfoxonium iodide (MeSOI) led to **40**, then treated with sodium iodide (NaI) followed by treatment with methanol under palladium-catalysis to give ganaxolone (**41**). The reported yield of the last stage was 90% [19].

### 2.6. Lutetium (^177^Lu) Vipivotide Tetraxetan

Lutetium (^177^Lu) vipivotide tetraxetan (**53**) (brand name Pluvicto^®^) is a urea-based inhibitor of the prostate-specific membrane antigen (PSMA). It is expected that the global sales forecast in 2026 will be $851 million [6].

Mechanism of action arises from lutetium-177 that delivers its beta-minus emission to PSMA-expressing cells [20].

Developed by Advanced Accelerator Applications, it has been authorized in March 2022 (23 March 2022) for the treatment of prostate-specific membrane antigen-positive metastatic castration-resistant prostate cancer where other therapies failed [3].

**Scheme 6:**
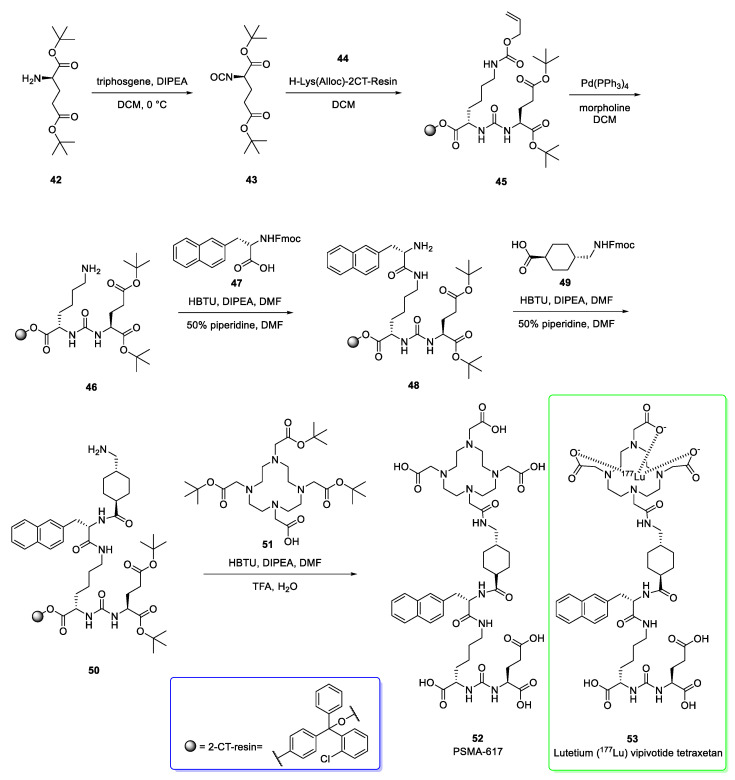
Synthesis of PSMA-617 (**52**) [22,23]. The structure of the 2-chloro-tritylresin (2-CT- resin) is reported as well (blue box). Lutetium (^177^Lu) vipivotide tetraxetan is shown.

Its structure is composed of PSMA-617, a human prostate-specific membrane antigen (PSMA)-targeting ligand, conjugated to the beta-emitting radioisotope lutetium, Lu 177 (^177^Lu) [21]. The synthetic pathway for PSMA-617 (Scheme 6) proceeds via solid-phase peptide chemistry [22,23]. The first step involves the conversion of the amino group of the bis-*tert*-butyl protected L-glutamate **42** to isocyanate **43** by means of triphosgene. Subsequently, a resin-immobilized (2-chloro-tritylresin) ε-allyloxycarbonyl protected lysine **44** was added to afford the urea **45**. The allyloxy protecting group was cleaved by using tetrakis(triphenylphosphine)palladium(0) (Pd(PPh_3_)_4_) and morpholine to give compound **46** which was condensed with Fmoc-3-(2-naphthyl)-L-alanine (Fmoc-2-Nal-OH, **47**) via HBTU activation and later treated with a solution of piperidine to afford the naphthyl-based pseudopeptide **48**. The free amino group of **48** was condensed with *trans*-4-(Fmoc-aminomethyl)cyclohexanecarboxylic acid **49** followed by deprotection of Fmoc to give **50**. The free aminomethyl group of **50** was eventually coupled with tri-*tert*-butyl 1,4,7,10-tetraazacyclododecane-1,4,7,10-tetraacetate [tris-(tert-but)DOTA, **51**] to provide PSMA-617 (**52**).

### 2.7. Oteseconazole

The antifungal oteseconazole (**67**, brand name Vivjoa^®^) was approved in April 2022 (26 April 2022) with the indication of reducing the incidence of recurrent vulvovaginal candidiasis (RVVC) in females with a history of RVVC who are not of reproductive potential [3].

Launched by Mycovia Pharmaceuticals [24], it is a tetrazole-based compound inhibiting the enzyme CYP51, known as 14α demethylase, leading to fungal destruction.

**Scheme 7:**
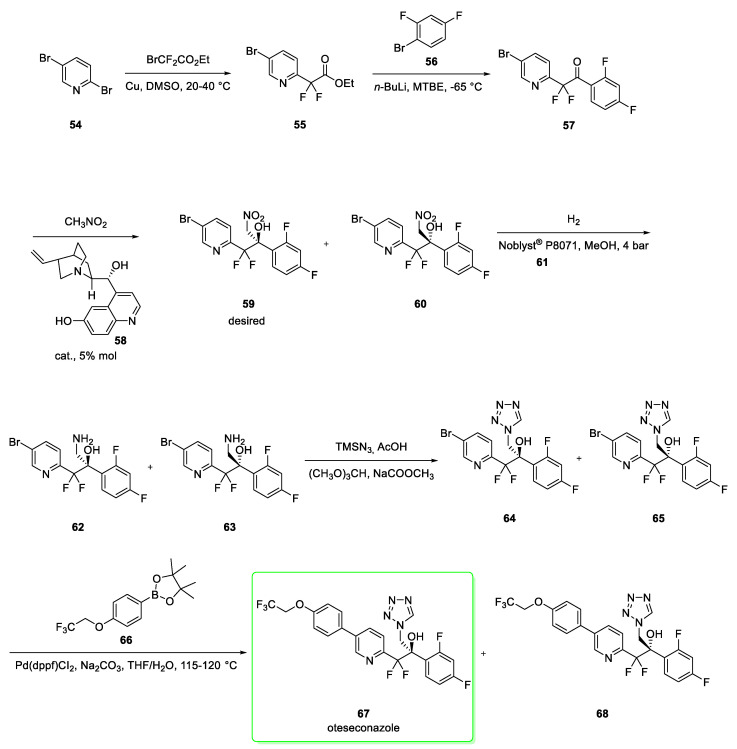
Synthesis of oteseconazole (**67**) [25,26].

Its synthesis (Scheme 7) [25,26] starts from the reaction between 2,5-dibromopyridine **54** and ethyl bromodifluoroacetate via a copper catalyzed nucleophilic addition to afford the ester **55**. This was subjected to nucleophilic attack of the lithiated l-bromo-2,4-difluorobenzene (**56**) at −65 °C to afford derivative **57**. The following Henry reaction provided the mixture of nitro alcohols **59** (with desired R chirality) and **60** (S enantiomer of **59**) in the presence of 5% mol of organocatalyst **58**. The enantiomeric ratio of the couple **59:60** was 90:10. The organocatalyst **58** proved to be the most effective in stereoselectivity among the many others tested. The subsequent catalytic hydrogenation (using the platinum catalyst Noblyst^®^ P8071, **61**) of the mixture of nitro alcohols led to the corresponding amines **62** and **63**. The conversion of **62** and **63** to the corresponding tetrazoles **64** and **65** was mediated by trimethylsilyl azide and trimethyl orthoformate in the presence of acetic acid. The last step involves a Suzuki coupling between the aryl bromide of 64 and 65 and the boronic ester 66 catalyzed by (diphenylphosphino) ferrocene] dichloropalladium (II) to generate the desired oteseconazole **67**. The authors claim that an enantioenriched (95.9%) form of **67** was obtained via diastereomeric recrystallisation with (*S*)-Camphor sulfonic acid [25].

### 2.8. Mavacamten

Mavacamten (75, brand name Camzyos^®^) was approved in April 2022 (28 April 2022) for the treatment of certain classes of obstructive hypertrophic cardiomyopathy [3]. It is an orally active cardiac myosin inhibitor developed by MyoKardia [27]. It is expected that the global sales forecast in 2028 will be $1.658 billion [28].

**Scheme 8:**
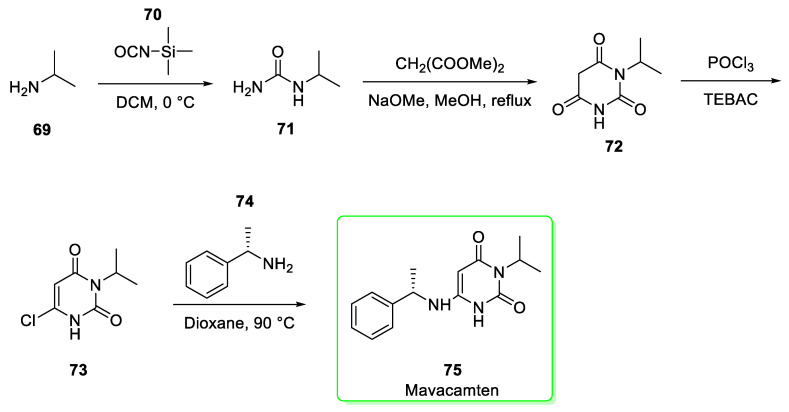
Synthesis of mavacamten (**75**) [29].

Its synthesis (Scheme 8) [29] starts from isopropylamine (**69**) which upon reaction with trimethylsilyl isocyanate **70** is converted to isopropylurea **71**. The reaction between **71** and dimethyl malonate in the presence of sodium methoxide as base gave 1-isopropyl barbituric acid **72**. The subsequent conversion to chloride derivative **73** is mediated by POCl_3_ using triethylbenzylammonium chloride (TEBAC) as phase transfer catalyst. The last stage is the nucleophilic addition of (S)-methylbenzylamine (**74**) to **73** in order to afford mavacamten **75**. Last step yield was 69% [29].

### 2.9. Vonoprazan (in Combination with Amoxicillin, and Clarithromycin)

Vonoprazam (in combination with amoxicillin and clarithromycin, brand name Voquezna^®^) was approved in May 2022 (3 May 2022) for the treatment of *Helicobacter pylori* infection [1]. It is expected that the global sales forecast in 2028 will be $869 million [28]. Launched by Takeda, it is a an orally bioavailable potassium-competitive acid blocker (P-CAB) [30]. P-CABs reversibly inhibit gastric acid secretion by competing with the K^+^ on the luminal surface, preventing the acid secretion [31]. The inhibition of gastric secretion combined to the antibacterial activities of amoxicillin and clarithromycin makes Voquezna^®^ a synergistically active “combo”.

**Scheme 9:**
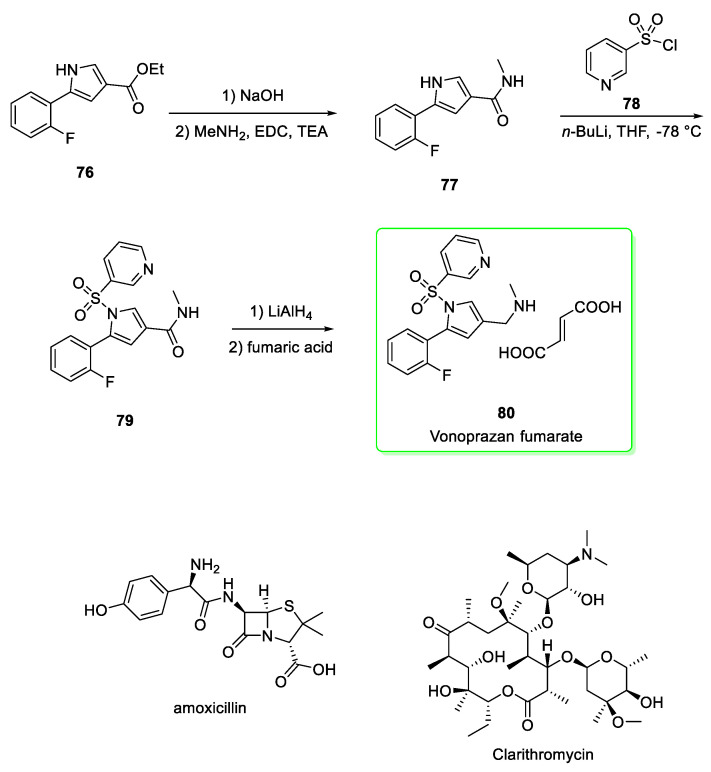
Synthesis of vonoprazan fumarate (**80**) [32]. The structure of amoxicillin and clarithromycin are reported for completeness in the lower part of the Scheme.

A very practical synthetic pathway (Scheme 9) for the preparation of vonoprazan fumarate [32] starts from the alkaline hydrolysis of the fluoroaryl-pyrrole **76** followed by the activation of carboxylic acid by 1-ethyl-3-(3-dimethylaminopropyl)carbodiimide (EDC) and the coupling with methylamine to afford amide **77**. The deprotonation of pyrrole-NH by *n*-butyllithium and the attack to pyridine-3-sulfonyl chloride **78** provided the sulfonamide **79**. The subsequent reduction of amide to amine, addition of fumaric acid and the recrystallization from methanol yielded vonoprazan fumarate **80**.

Interestingly, no product was observed if other bases such as NaH, *t*-BuOK, or TEA were used instead of *n*-butyllithium in the sulfonamidation step. The authors highlight that other reducing agents such as BH_3_, Red-Al and NaBH_4_-BF_3_ were ineffective in the reduction stage from amide to amine. Overall yield was 41.3% [32].

### 2.10. Tapinarof

Tapinarof (brand name Vtama^®^) was approved in May 2022 (23 May 2022) for the treatment of plaque psoriasis [1]. It is an aryl hydrocarbon receptor (AhR) agonist that is being developed by Dermavant Sciences Inc. (a subsidiary of Roivant Sciences Inc., Basel, Switzerland) [33]. It is a naturally derived small molecule produced by bacterial symbionts of entomopathogenic nematodes [34].

**Scheme 10:**
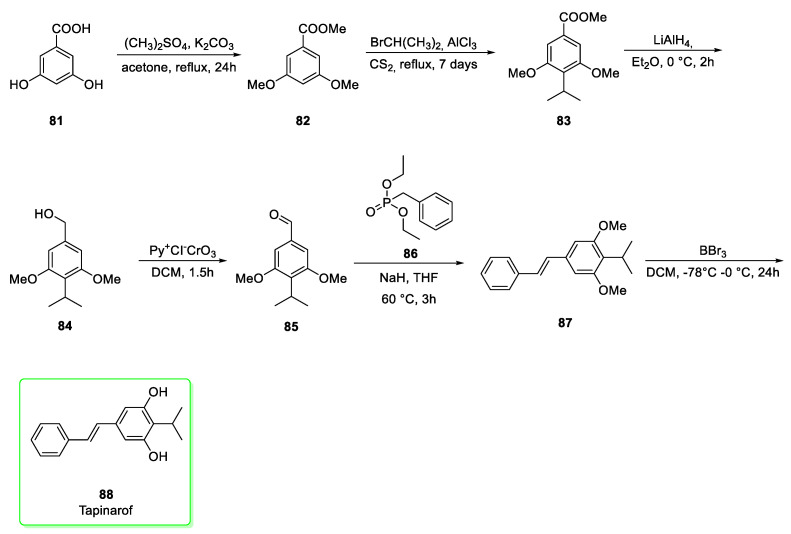
Synthesis of tapiranof (**88**) [35].

Tapinarof is synthesized (Scheme 10) [35] starting from the commercially available 3,4-dihydroxybenzoic acid **81**. The methylation step by using dimethyl sulfate afforded the trimethylated derivative **82**. The subsequent Friedel–Crafts alkylation of **82** with isopropyl bromide in carbon disulfide afforded the isopropyl intermediate **83**. Ester of **83** was reduced to alcohol **84** by LiAlH_4_ and later oxidized to aldehyde (**85**) by using pyridinium chlorochromate. The Horner–Wadsworth–Emmons (HWE) reaction between **85** and diethyl benzylphosphonate ester (**86**) yielded the *E*-alkene **87**. The demethylation step mediated by BBr_3_ gave tapinarof (**88**). Last step yield was 95%.

### 2.11. Deucravacitinib

Deucravacitinib (brand name Sotyktu^®^) was approved in September 2022 (9 September 2022) and for the treatment, as in the case of tapinarof, of plaque psoriasis [1]. It is expected that the global sales forecast in 2026 will be $1.12 billion [36].

It is a deuterated drug launched by Bristol Meyers Squibb [35] selectively targeting tyrosine kinase 2 (TYK2), a member of the Janus family of kinases (JAK). The incorporation of deuterium, the authors say, is important, to “slow down the production of an otherwise promiscuous metabolite”. Metabolic stability is an important parameter to take into account and novel deuterium-based drugs can appear in the next years. Moreover, C–D bonds are shorter and at times more resistant than C–H bonds [37,38].

**Scheme 11:**
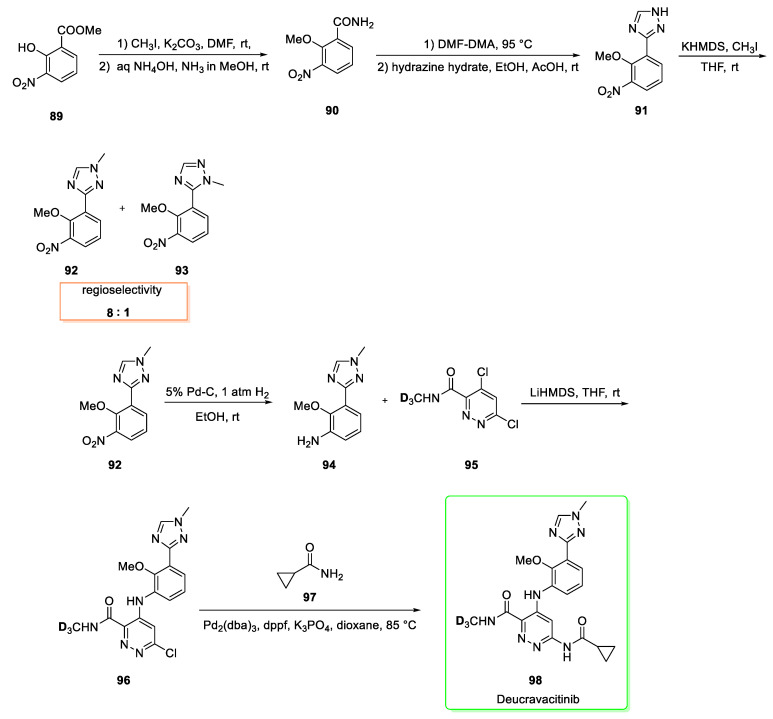
Synthesis of deucravacitinib (98) [38].

Its synthesis (Scheme 11) [38] starts from methyl-2-hydroxy-3- nitrobenzoate **89** which is firstly methylated with methyl iodide and later converted into amide by ammonolysis to afford the intermediate **90**. The treatment of **90** with DMF-DMA followed by condensation with hydrazine hydrate yielded the triazole **91** which was then methylated in presence of methyl iodide and potassium hexamethylsilazide to generate compound **92** in a good regioselectivity (8:1) over compound **93**.

Pure compound **92** was subjected to catalytic hydrogenation (Pd-C, H_2_) to give the substituted aniline **94** which was then mixed with the pyridazine **95** [39] in the presence of lithium hexamethyldisilazide as a base to afford the diaryl aniline **96**. The last stage involves a palladium-catalyzed Buchwald−Hartwig reaction between **96** and cyclopropyl amide **97** to form Deucravacitinib (**98**). The reaction was optimized and it was found that a combination of 1,1′-bis(dicyclohexylphosphino)ferrocene (dppf), tris(dibenzylideneacetone)dipalladium(0) [Pd_2_(dba)_3_] and aqueous potassium triphosphate resulted in a better yield and milder conditions if compared to the triad XantPhos/Pd_2_(dba)_3_/Cs_2_CO_3_. Last step yield was 76%.

### 2.12. Gadopiclenol

Approved in September 2022 (21 September 2022), gadopiclenol is used to detect and visualize lesions, together with Magnetic Resonance Imaging (MRI), with abnormal vascularity in the central nervous system and the body [1].

Gadopiclenol (brand name Elucirem^®^ [1]) is a macrocyclic gadolinium-based contrast agent (GBCA) having high relaxivity properties, which was designed to increase lesion detection and characterization by magnetic resonance imaging [40]. It was launched by Bracco [41].

**Scheme 12:**
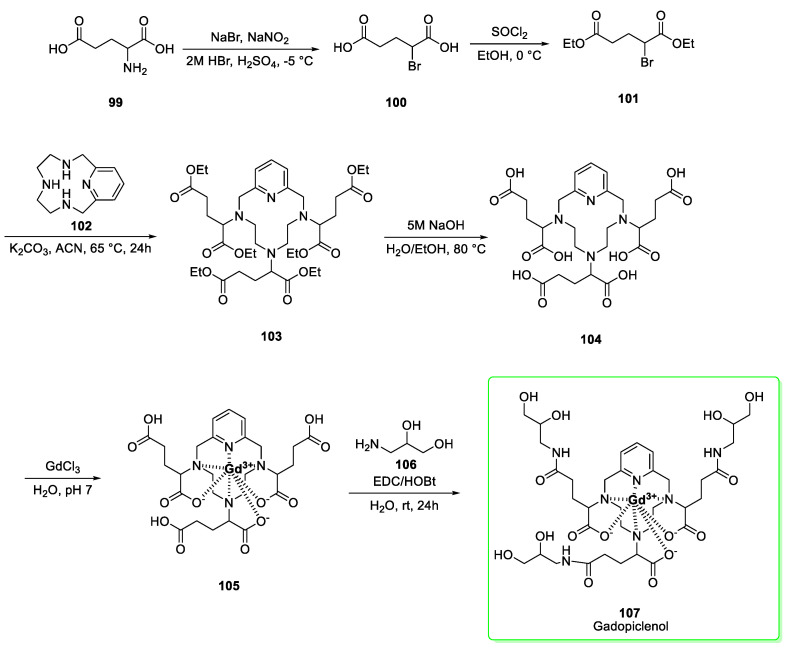
Synthesis of gadopiclenol isomeric mixture (**107**) [42].

Its synthetic preparation (Scheme 12) [42] originates with racemic glutamic acid. The amino group is diazotated and attacked by bromide to afford derivative **100**. The conversion of **100** to the diethyl-ester **101** was obtained via using thionyl chloride in ethanol. The subsequent nucleophilic substitution between **101** and 3,6,9-triaza-1(2,6)-pyridinacyclodecaphane (**102**) yielded the intermediate **103** which was subjected to alkaline hydrolysis to afford the exa-acid **104**. The following complexation with gadolinium by adding gadolinium (III) chloride afforded the intermediate **105** which was converted to the isomeric gadopiclenol (**107**) by condensation (EDC/HOBt conditions) of the free carboxylic acid groups with isoserinol (**106**). Last step yield was 78%.

### 2.13. Oomidenepag Isopropyl

Oomidenepag isopropyl (brand name Omlonti^®^ [1]) was approved in September 2022 (22 September 2022) with the indication of reducing elevated intraocular pressure in patients with open-angle glaucoma or ocular hypertension [1]. It is expected that the global sales forecast in 2026 will be $121 million [36].

It is a selective prostaglandin E2 receptor agonist with a non-prostaglandin structure that is being developed by Ube Industries and Santen Pharmaceutical in Japan, Singapore and the USA [43]. Oomidenepag isopropyl is a prodrug being converted in vivo into its active form, oomidenepag [44].

**Scheme 13:**
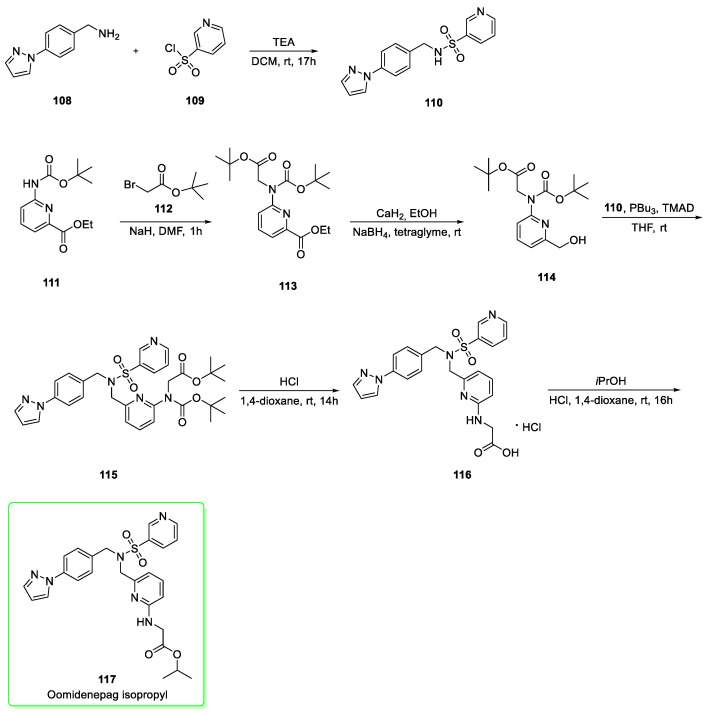
Synthesis of oomidenepag isopropyl (117) [45,46,47].

Its synthesis (Scheme 13) [45,46,47] originates from the preparation of the pyrazole synthon **110**. This was prepared by nucleophilic addition of substituted benzylamine **108** to pyridine-3-sulfonyl chloride (**109**) in the presence of triethylamine. In parallel, the initial alkylation of BOC-protected aniline **111** with tert-butyl 2-bromoacetate (**112**) was carried out in presence of sodium hydride. The resulting **113** which was reduced (calcium hydride and NaBH_4_ conditions) at the ester functionality to afford alcohol **114**. The subsequent Mitsunobu reaction between **110** and **114** mediated by tributylphosphine and tetramethylazodicarboxamide (TMAD) afforded the substituted sulfonamide **115**. The removal of BOC-protecting group of **115** afforded **116** with the simultaneous hydrolysis at the tert-butyl ester moiety. The final stage is given by the esterification with isopropanol to afford oomidenepag isopropyl (**117**).

### 2.14. Taurursodiol (in Combination with Sodium Phenylbutyrate)

Approved (brand name Relyvrio^®^ [1]) on 29 September 2022 for amyotrophic lateral sclerosis (ALS) in combination with sodium phenylbutyrate, taurursodiol is a tauro-bile acid conjugate. It is expected that the global sales forecast in 2026 will be $1.095 billion [36]. Combination product Relyvrio^®^ is postulated to have a synergistic effect that can reduce neuronal death by simultaneous inhibition of endoplasmic reticulum and mitochondrial stress [48].

**Scheme 14:**
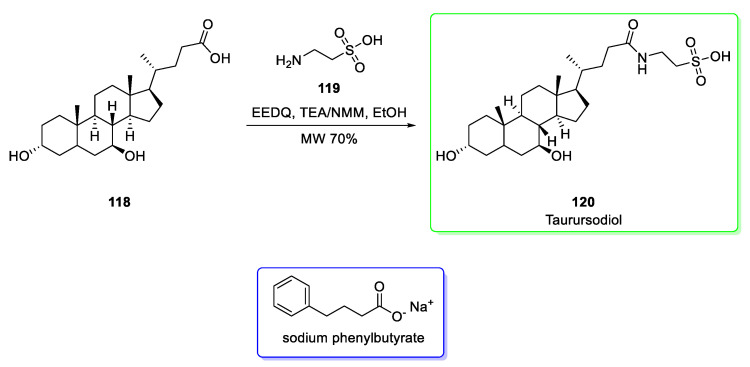
Synthesis of taurursodiol (**120**) obtained via MW from ursodeoxycholic acid [51]. Structure of sodium phenylbutyrate is reported (blue box).

Taurursodiol can be obtained from ursodeoxycholic acid (**118**). This latter is naturally present in bears’ livers and related carnivores [49]. Otherwise, semisynthetic ursodeoxycholic acid is obtained in 30% of yield [50]. A convenient synthesis of taurursodiol (Scheme 14) proceeds via microwave [51]. The condensation between the carboxylic acid of ursodeoxycholic acid (**118**) and taurine (**119**) by adding *N*-ethoxycarbonyl-2-ethoxy-1,2-dihydroquinoline (EEDQ), triethylamine (TEA) or *N*-Methylmorpholine (NMM) in ethanol under microwave afford taurursodiol (**120**). Last stage yield was 67%.

### 2.15. Futibatinib

Approved on 30 September 2022 for the treatment of intrahepatic cholangiocarcinoma harboring fibroblast growth factor receptor 2 (FGFR2) gene fusions or other rearrangements [1], futibatinib (brand name Lytgobi^®^ [1]) is a highly selective irreversible fibroblast growth factor receptor (FGFR1–4) inhibitor [52]. Constitutive FGFR signaling can support the proliferation and survival of malignant cells.

**Scheme 15:**
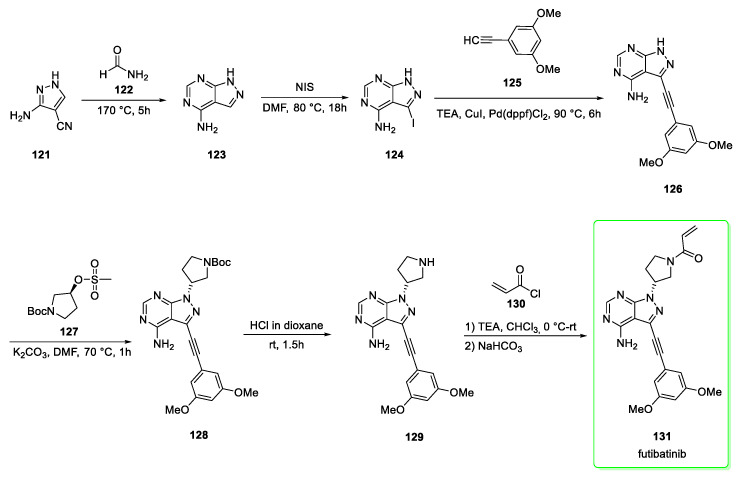
Synthesis of futibatinib (131) [53,54].

Its structure contains a pyrazolo-pyrimidine scaffold and the synthesis (Scheme 15) [53,54] starts from 3-amino-1*H*-pyrazole-4-carbonitrile (**121**) which is transformed into pyrazolo-pyrimidine **123** by addition of formamide (**122**). The following conversion to iodo-derivative **124** was mediated by *N*-iodosuccinimide (NIS) in DMF. Sonogashira coupling [triethylamine, cuprous iodide and diphenylphosphino)ferrocene]dichloropalladium (II)] between halide **124** and alkyne **125** provided the derivative **126**. The subsequent nucleophilic attack (resulting in inversion of configuration) of **126** to mesyl-protected alcohol **127** afforded the pyrrolidine derivative **128** which was subjected to BOC-removal by addition of HCl to give **129**. The final step involves another nucleophilic attack (acylation) of **129** to acryloyl chloride (**130**) followed by neutralization with sodium bicarbonate to yield futibatinib (**131**).

## 3. Sulfonamide: An Historical Re-Occurring Moiety in the FDA-Approved Drug List

Sulfonamide-containing drugs (four entities) are arising among the approved molecules in 2022. Sulfonamides were among the first examples of carboxylic acid isosteres to show utility in drug design [55].

**Figure 1:**
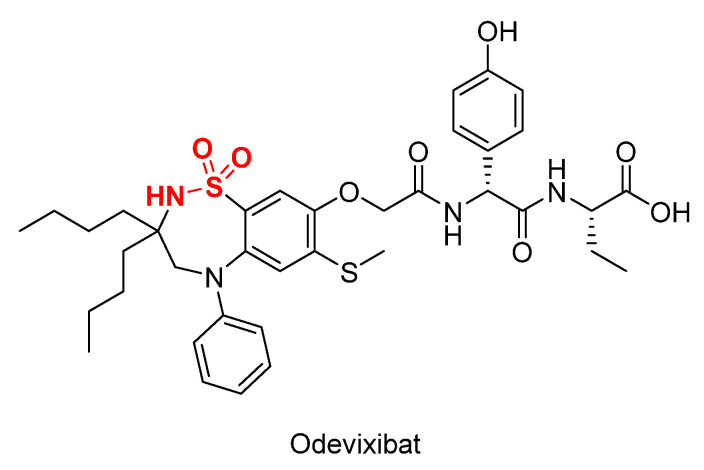
Chemical structure of odevixibat. The sulfonamide moiety is shown in red.

In the previous year (2021), only odevixibat (brand name Bylvay^®^, Figure 1), a drug containing cyclic sulfonamide, was approved for the treatment of pruritus [56], while in 2020 no sulfonamide-containing drugs were approved by FDA [57].

**Figure 2:**
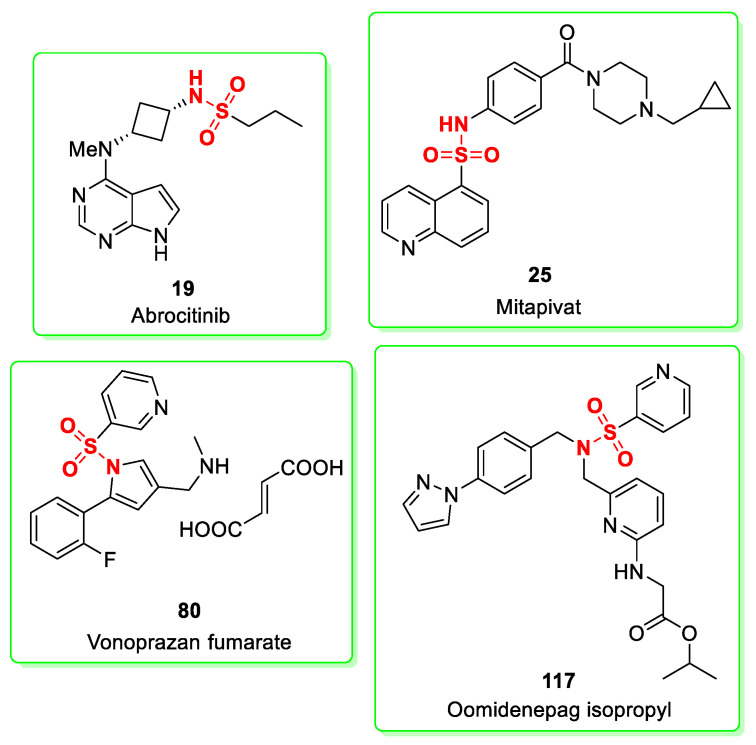
Chemical structure of approved sulfonamides in 2022. Abrocitinib (**19**), mitapivat (**25**), vonoprazan fumarate (**80**) and oomidenepag isopropyl (**117**). The sulfonamide moieties are shown in red.

The approved sulfonamides in 2022 are a Janus kinase inhibitor (abrocitinib, **19**), a pyruvate kinase enzyme activator (mitapivat, **25**), a potassium-competitive acid blocker (P-CAB) represented by vonoprazan (vonoprazan fumarate, **80**) and a selective prostaglandin E2 receptor agonist (oomidenepag isopropyl, **117**). None of these molecules (Figure 2) are cyclic sulfonamides (also known as sultams) differing from odevixibat approved in 2021. Sultams are often incorporated into the target molecules as a stable lactam equivalent [58].

**Figure 3:**
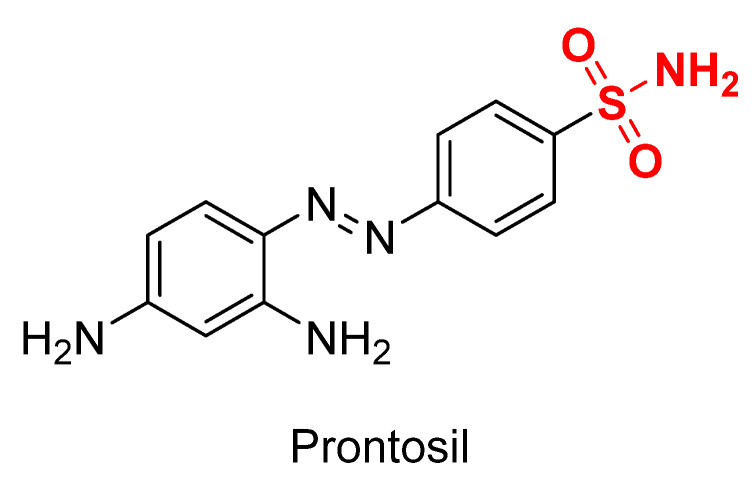
Chemical structure of prontosil. The sulfonamide moiety is shown in red.

Historically, the first sulfonamide drug was prontosil rubrum (Figure 3). This compound was first synthesized by Bayer chemists Josef Klarer and Fritz Mietzsch as part of a research program designed to find dyes that might act as antibacterial drugs in the body [59]. It was the year 1932. Later on, sulfonamides have been broadly used as antibacterial agents given their similarity to *p*-aminobenzoic acid (PABA) in the synthesis of folic acid which is essential for the further production of DNA in the bacteria [60]. Another well-known explored field has been the use of sulfonamides and isosteres as inhibitors of metalloenzyme carbonic anhydrase [61].

Interestingly, none of the sulfonamides approved by FDA in 2022 belong to the last two groups of inhibitors, creating novel opportunities in hitting novel and diverse targets.

Moreover, a key-feature that would help in the design of novel bioactive compounds is the fact that sulfonamides possess high hydrolytic stability [62].

## 4. Concluding Remarks

In this work, all small molecule drugs that were approved by the FDA in 2022 were discussed. For each compound, the biological activity and the chemical synthesis were provided. It appears that sulfonamide-containing drugs are dominating the year 2022 with four molecules approved. Their physicochemical and pharmacodynamic properties still play an important role many years after the discovery of the first sulfonamides endowed with antibacterial activities.

Deuterium-containing drug deucravacitinib represents, on the other hand, an example of novel approaches in drug discovery incorporating an unusual isotope to provide drug stability.

The discovery of pyrimidinedione mavacamten for the treatment of cardiomyopathy and the huge forecasted sales (Table S1) for this molecule stress the importance of finding new active molecules in the field of cardiovascular diseases.

The combination of drugs, for example vonoprazan (in combination with amoxicillin and clarithromycin) and taurursodiol (in combination with sodium phenylbutyrate) is still a valid weapon for the treatment of unmet medical diseases.

The 15 FDA-approved small molecules (Figure S1) in 2022 are examples of classical and innovative drug discovery approaches which are paving the way for future, exciting approvals.

## Data Availability

Not applicable.

## Supplementary Materials

The following supporting information can be downloaded at: https://www.mdpi.com/article/10.3390/pharmaceutics14112538/s1, Table S1: Table describing the names of the 15 molecules approved by FDA in 2022. Figure S1: Global sales forecast in 2026 (* 2028 for some drugs).
