# Supplementary material for: FDA-Approved Small Molecules in 2022: Clinical Uses and Their Synthesis
Source: Pharmaceutics. 2022 Nov 21;14(11):2538. doi: 10.3390/pharmaceutics14112538 (PMC9695118; doi:10.3390/pharmaceutics14112538)
Supplement: Supplementary file 1 [file pharmaceutics-14-02538-s001.zip › pharmaceutics-2028373-supplementary.pdf]

pharmaceutics

MDPI

SUPPORTING INFORMATION

FDA-Approved Small Molecules in 2022: Clinical Uses and Their Synthesis

Davide Benedetto Tiz, Luana Bagnoli, Ornelio Rosati, Francesca Marini, Claudio Santi and Luca Sancineto

Content:

Table describing the names of the 15 molecules approved by FDA in 2022 (**Table S1**)	**Pages 2-5**
Global sales forecast in 2026 (*2028 for some drugs) (**Figure S1**)	**Page 6**
References	**Page 7**

pharmaceutics

MDPI

Name	Chemical structure	Therapeutic indication
**Daridorexant**		insomnia
**Abrocitinib**		atopic dermatitis (AD)
**Mitapivat**		hereditary hemolytic anemias
**Pacritinib**		high-risk primary or secondary myelofibrosis
**Ganaxolone**		seizures in cyclin-dependent kinase-like 5 deficiency disorder

pharmaceutics

MDPI

**Lutetium (^{177}Lu) vipivotide tetraxetan**		specific membrane antigen-positive metastatic castration-resistant prostate cancer
**Oteseconazole**		recurrent vulvovaginal candidiasis (RVVC) in females
**Mavacamten**		obstructive hypertrophic cardiomyopathy
**Vonoprazan**		*Helicobacter pylori* infection (in combination with amoxicillin and clarithromycin)
**Tapiranof**		plaque psoriasis

pharmaceutics

**Deucravacitinib**		plaque psoriasis
**Gadopiclenol**		detect and visualize lesions, together with MRI, with abnormal vascularity in the central nervous system and the body
**Oomidenepag isopropyl**		reducing elevated intraocular pressure in patients with open-angle glaucoma or ocular hypertension
**Tauroursodiol**		amyotrophic lateral sclerosis (ALS) in combination with sodium phenylbutyrate

**Futibatinib**		intrahepatic cholangiocarcinoma

Table S1. Table describing the names of the 15 molecules approved by FDA in 2022. Their structures and therapeutic use are provided as well [1].

pharmaceutics

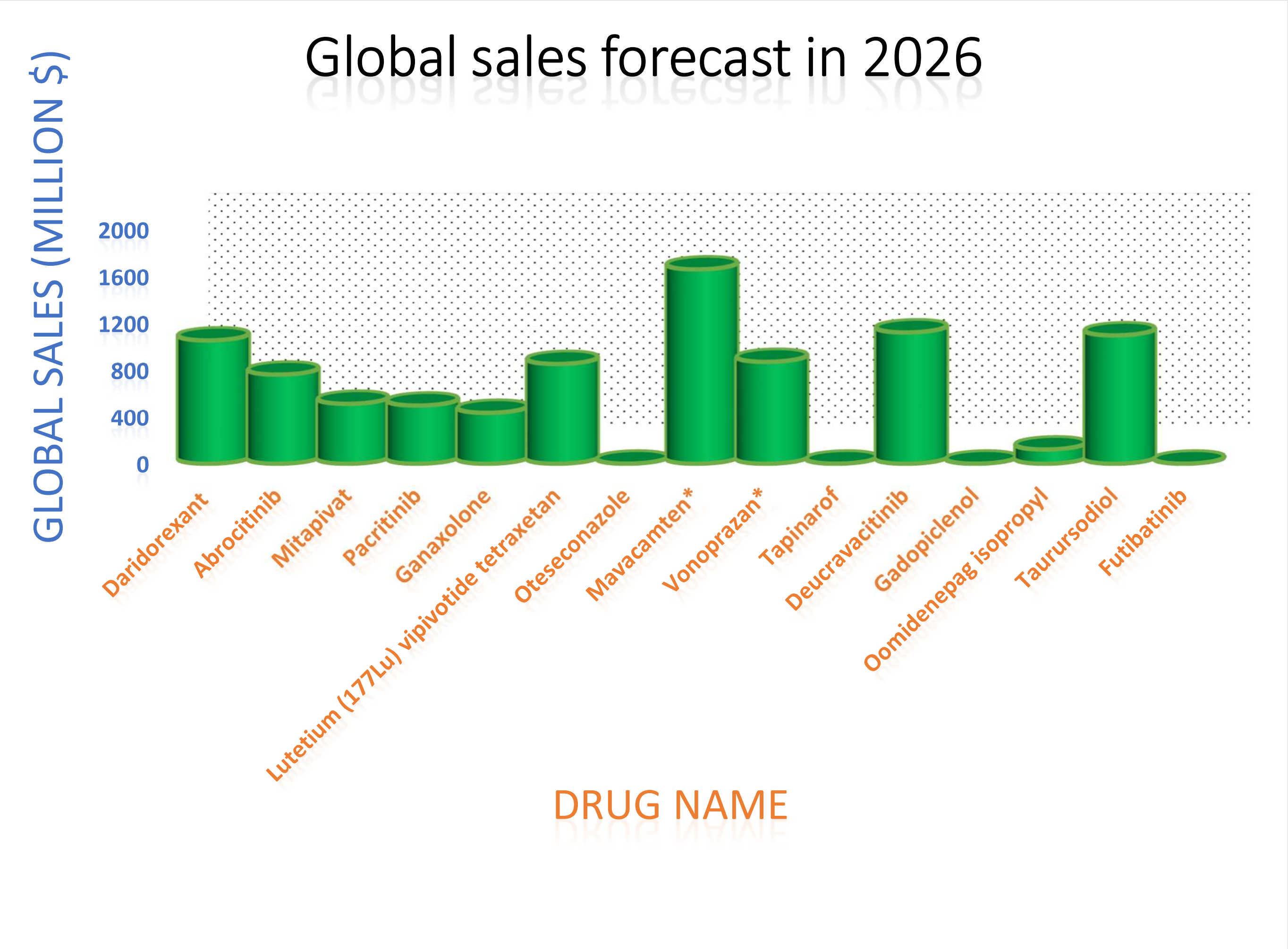

Figure S1. Forecasted global sales for 2022 FDA-approved drugs (in million $ in 2026). Values for mavacamten and vonoprazan are meant for 2028. Data for oteseconazole, tapinarof, gadopiclenol, and futibatinib were not available [2–4].

References

1. Novel Drug Approvals for 2022|FDA. Available online: https://www.fda.gov/drugs/new-drugs-fda-cders-new-molecular-entities-and-new-therapeutic-biological-products/novel-drug-approvals-2022 (accessed on 24 October 2022).
2. Urquhart, L. FDA New Drug Approvals in Q1 2022. *Nat. Rev. Drug. Discov.* **2022**, 21, 329–329, doi:10.1038/d41573-022-00063-9.
3. Urquhart, L. FDA New Drug Approvals in Q2 2022. *Nat. Rev. Drug. Discov.* **2022**, *21*, 550–550, doi:10.1038/d41573-022-00123-0.
4. Brown, A. FDA New Drug Approvals in Q3 2022. *Nat. Rev. Drug Discov.* **2022**, doi:10.1038/d41573-022-00174-3.
